# Concurrent Urinary Bladder Paraganglioma and Adrenal Phaeochromocytoma With Succinate Dehydrogenase-B Mutation

**DOI:** 10.7759/cureus.17350

**Published:** 2021-08-21

**Authors:** Bayan Hafiz, Omar Buksh, Adel Alammari, Ahmed Khogeer, Samirah Alturkistani, Wafaey Gomaa, Jaudah Al-Maghrabi

**Affiliations:** 1 Department of Anatomic Pathology, King Abdulaziz Medical City, Jeddah, SAU; 2 Department of Urology, King Faisal Specialist Hospital and Research Centre, Jeddah, SAU; 3 Department of Radiology, King Faisal Specialist Hospital and Research Centre, Jeddah, SAU; 4 Department of Pathology, Faculty of Medicine - King Abdulaziz University, Jeddah, SAU; 5 Department of Pathology, Faculty of Medicine - Minia University, Al Minia, EGY

**Keywords:** phaeochromocytoma, paraganglioma, sdhb deficiency, adrenal, bladder

## Abstract

Phaeochromocytoma (PHEO) is a neoplasm that arises from chromaffin cells present in the adrenal medulla. The counterpart of the PHEO extra-adrenal is termed paraganglioma (PGL). The urinary bladder PGL is a rare tumour, and it accounts for less than 0.06% of all bladder tumours. In this report, we discuss a case of a young female who presented with symptoms of headache, dizziness, palpitations, and high blood pressure. After workup, she was diagnosed with concurrent urinary bladder PGL and adrenal PHEO, and the genetic study of the whole exon sequence indicated the presence of succinate dehydrogenase-B (SDHB) mutation. Both tumours were treated surgically; however, the patient ultimately developed recurrence, rapid progression, and metastasis. All secondary modalities were unsuccessful, and the patient was referred for palliative treatment and eventually lost to follow-up.

PGL should be included in the differential diagnosis of bladder tumours, and testing for SDHB gene mutations should be considered in all urinary PGLs. Therefore, these patients need follow-up and genetic counselling.

## Introduction

Phaeochromocytoma (PHEO) and paraganglioma (PGL) are tumours of neuroendocrine origin. PGL arises from extra-renal chromaffin tissue and occurs in the head and neck, abdomen, and pelvis [1]. PGL can involve any tissue arising from embryonic chromaffin cells, including the sympathetic nerve plexus of the detrusor muscle, retroperitoneally along the sympathetic chains, and in the carotid bodies and organs of Zuckerkandl near the inferior mesenteric artery [2]. PGL of the urinary bladder is extremely rare and accounts for less than 0.06% of all bladder tumours, with a wide group range effect, and its most common location is the dome followed by the trigone of the bladder [3]. PHEO and PGL are classified into functioning (chromaffin) and non-functioning (non-chromaffin) tumours based on catecholamine secretion [4]. PHEO and PGL can be part of many hereditary syndromes like Hippel-Lindau, multiple endocrine neoplasias, familial PGL syndromes, and succinate dehydrogenase (SDH) syndromes. SDH has many subsets (A, B, C, and D), and the common gene subset associated with familial PGL syndrome is the SDHB gene. The urinary PGL has two forms: germline and sporadic mutation. The germline form has an aggressive behaviour and is associated with SDHB mutation [5,6].

We present a case of a female patient diagnosed with urinary bladder PGL and adrenal PHEO concurrently. The genetic study showed that she had an SDHB gene mutation. We also engage in a literature review of the cases of urinary PGL that had adrenal PHEO and analyse its association with SDHB gene mutation.

## Case presentation

**Figure 1:**
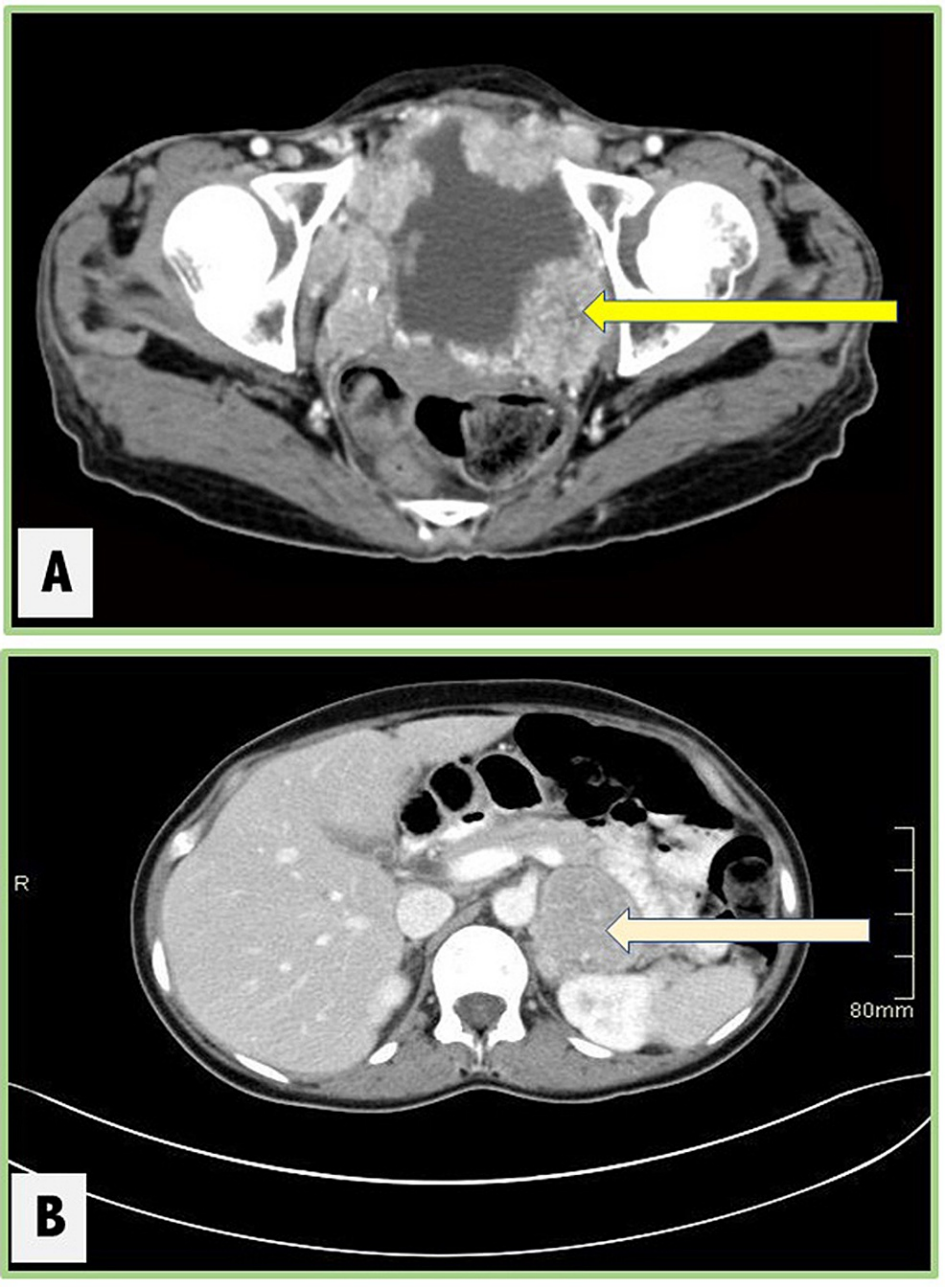
Radiology The images show a 5.6 x 5-cm left retroperitoneal mass inseparable from the adrenal gland and an 8.2 x 6.4-cm urinary bladder mass protruding into the lumen (arrows)

**Figure 2:**
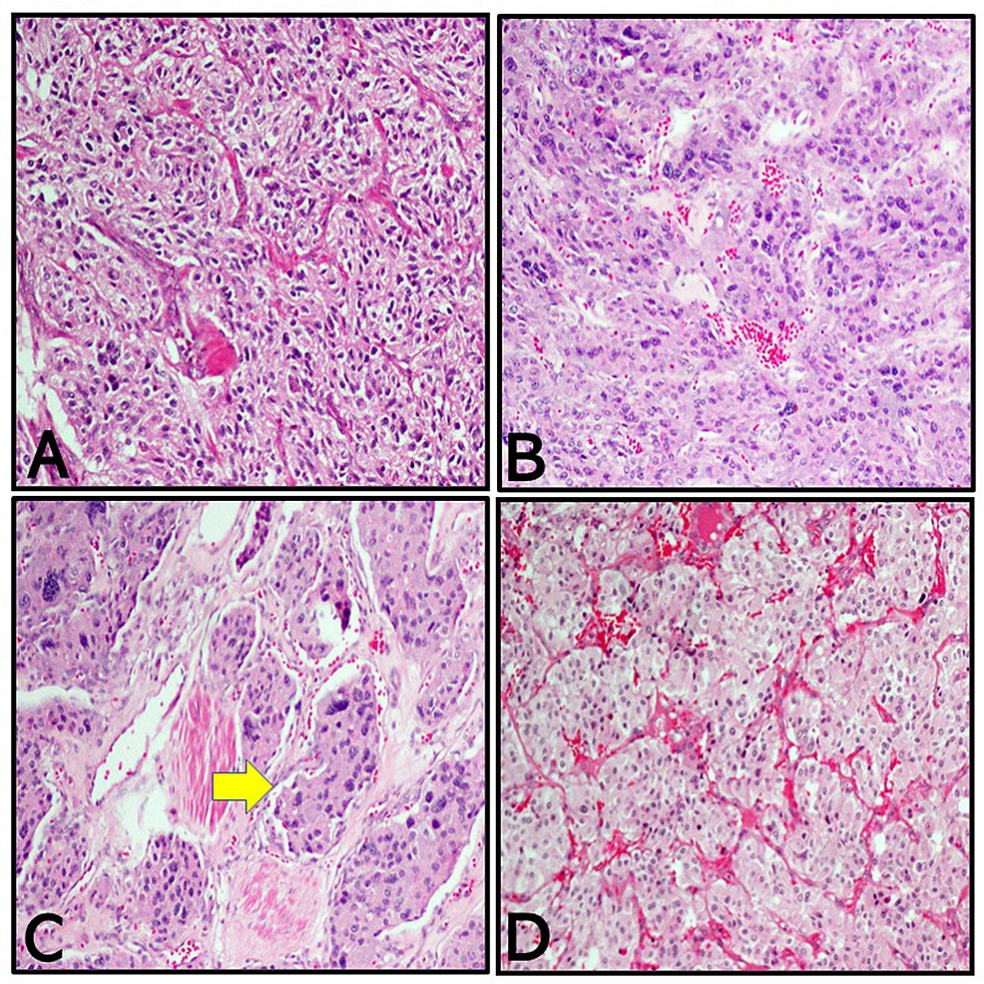
H&E Prominent nuclear atypia, vascular invasion, and frequent mitotic figures are seen H&E: hematoxylin and eosin

A 36-year-old female presented to the emergency room with severe headache and palpitation. She had a history of sweating, fatigue, and hypertension. Urine (24 hours) chemistry showed a normetanephrine level of 84.7 umol/L (normal range: 0-3 umol/L). Other endocrine tests were negative. CT scan demonstrated a 5.6 x 5-cm left retroperitoneal mass inseparable from the adrenal gland and an 8.2 x 6.4-cm urinary bladder mass protruding into the lumen (Figure 1). The nuclear scan noted intensely increased metaiodobenzylguanidine (MIBG) uptake in the left adrenal gland consistent with malignancy. Also, the urinary bladder showed increased tracer uptake consistent with metastases. The patient was referred to urological surgery, and a partial cystectomy (the patient refused radical cystectomy) was performed and the bladder mass was sent for histopathological examination. Grossly, the specimen was received fragmented, measuring 8.2 x 6 cm in aggregate. On microscopy, there was a neoplastic growth formed of round to oval cells with abundant eosinophilic or basophilic cytoplasm and featuring nesting and trabecular (zellballen) pattern within a prominent vascular network. Prominent nuclear atypia, vascular invasion, and frequent mitotic figures were seen (Figure 2).

**Figure 3:**
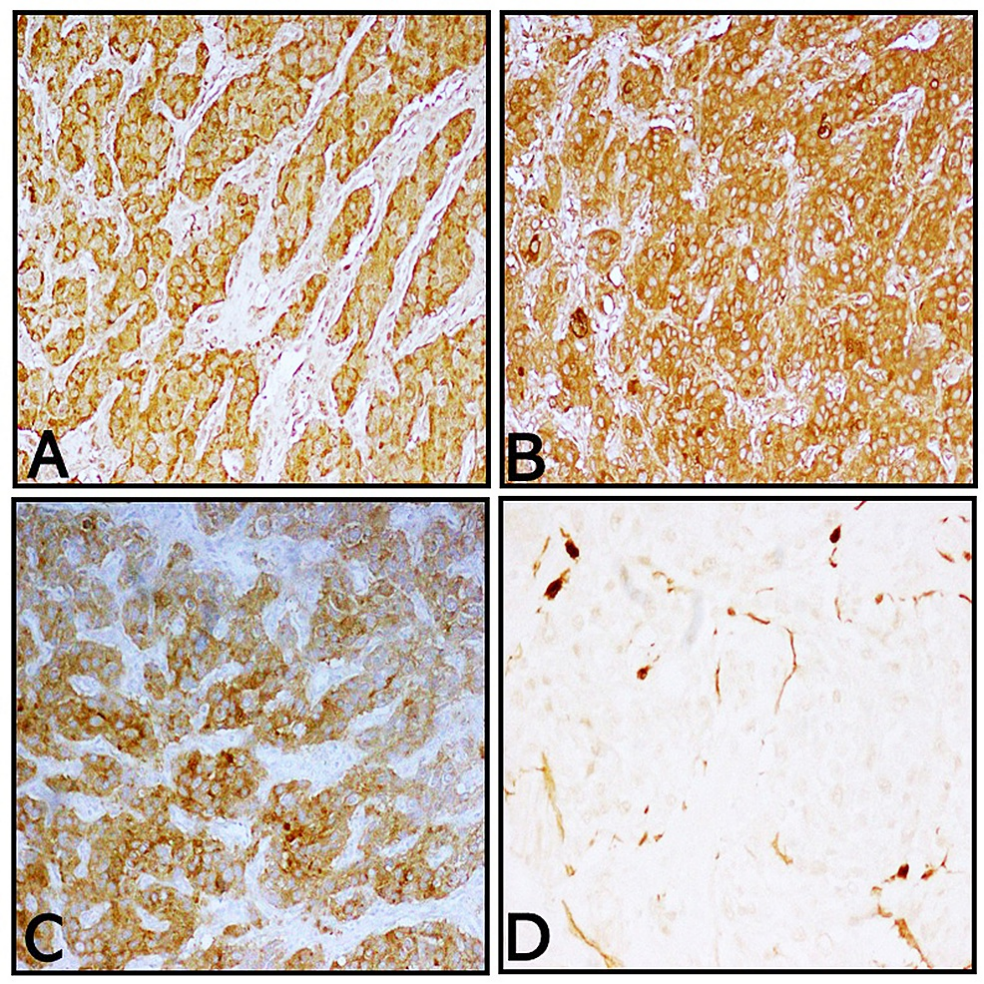
Immunohistochemistry The tumour cells showed strong diffuse positive for synaptophysin and chromogranin. The S100 was positive in sustentacular cells

The margins of excision were positive. A panel of immunohistochemistry was done including synaptophysin, chromogranin, neuron-specific enolase (NSE), and S100. The tumour cells showed strong diffuse positive for synaptophysin and chromogranin. The S100 was positive in sustentacular cells. Representative sections are shown in Figure 3.

The final diagnosis was urinary PGL. A genetic study was requested to detect mutation in all axon, axon-intron of SDHB, SDHC, and SDHD genes. The results confirmed the presence of heterozygosity for the following mutation in the SDHB gene: Exon 3, c.268c>T, p.Arg90x (p.R90X), which was consistent with the diagnosis of PGL-PHEO, a syndrome. Based on histopathological findings, the patient was strongly advised that a total cystectomy was needed. The patient refused and was subsequently discharged and given phenoxybenzamine, labetalol, and atenolol. Follow-up CT showed resolution of the bladder mass and interval increase in the size of the adrenal mass.

Six weeks after the initial procedure, the patient was admitted for laparoscopic adrenalectomy and was counselled on the possibility of converting to open, given the size of the adrenal mass. However, the patient underwent uneventful laparoscopic adrenalectomy with smooth postoperative recovery. On gross examination, the specimen was formed of multiple fragments of grey tan soft tissue measuring 8.2 x 6.2 cm in aggregate. The microscopic features were the same in morphology and immunohistochemistry of the bladder mass and the final diagnosis was adrenal PHEO. Postoperatively, her antihypertensive medication requirement decreased to zero, and she was discharged home in good general condition. On all subsequent radiographic images, there was no recurrence or residual disease at the adrenal bed. The patient had urinary bladder local recurrence and lung metastasis after 18 months. Eight months later, the patient showed progression of the bladder mass with haematuria and increased size of pulmonary metastasis. The patient refused any surgical intervention. The patient was given multiagent chemotherapy for eight cycles with cyclophosphamide, vincristine, and dacarbazine (CVD) with palliative intent. However, a year later, there was a progression of the bladder mass, pelvic adenopathy, and mild right-sided hydroureteronephrosis. Another year later, multiple CT evaluations were performed, which showed the progression of metastatic disease. The patient was scheduled to receive radioiodine I-123 MIBG treatment and she received two cycles of radioactive iodine MIBG. CT scans following the treatment showed further disease progression. Pazopanib was given as a salvage option in 2018. The patient continued with palliative care to control symptoms until she was lost to follow-up shortly afterward.

## Discussion

PGL arises in sympathetic and parasympathetic ganglia. PGL of sympathetic origin secretes catecholamine and presents with symptoms like headache, hypertension, and palpitations, and is usually located in the abdomen and trunk [7]. Primary urinary PGL is a rare tumour among the bladder tumours (0.05%), and it was first described by Zimmerman et al. in 1953 [8]; it accounts for 1% of all cases of PHEO [9]. The disease course of PHEO is usually benign but requires life-long follow-up due to the inability to predict metastatic potential by histology. Bladder PGLs behave in a similar manner. Metastatic disease is usually treated with palliative intent, chemotherapy being the current mainstay [2]. There are no well-established criteria to determine the malignant behaviour of PGLs. However, the World Health Organisation has defined malignant PGLs based on the presence of metastasis only [10]. PHEOs are neuroendocrine tumours arising from chromaffin tissues of the adrenal gland [1].

It is unusual to see PHEO/PGLs concurrently in one patient. In a study series by O’Riordain et al., 10 patients had adrenal PHEO [synchronous and metachronous; six showed sporadic mutation, and four presented familial diseases such as von Hippel-Lindau disease and multiple endocrine neoplasia type IIb (MEN-IIb)] [11]. Kapetanakis et al. have reported an old patient with a large retroperitoneal mass diagnosed as PGL with a microscopic finding of PHEO in ipsilateral adrenal glands without any connection between both tumours [12]. The present case was a unique one, characterised by simultaneous presentation of PHEO and PGL, which indicated genetic testing. A review of the literature showed no similar cases with a concurrent disease of the bladder and adrenal.

Multiple genes are associated with PHEO/PGLs with varying modes of inheritance and varying significance clinically. Germline and somatic mutations in RET, von Hippel-Lindau, and SDH have been studied [2,13]. Mutations affecting subunits of the SDH gene result in the accumulation of succinate and reactive oxygen species [2]. The SDH gene has four subunits: SDHA, SDHB, SDHC, and SDHD. Mutation of SDHD is associated with familial PGL syndrome. Patients present with sympathetic extra-adrenal PGL of the head and neck and occasionally unilateral PHEO. Karasek et al. have proposed recommendations for instances when genetic testing is indicated based on tumour location, catecholamine production, and histological evaluation [13].

A PHEO-PGL syndrome is a hereditary disease. PGL is associated with this syndrome caused by germline mutations in SDH [14]. Studies have demonstrated the association between PGLs in PHEO/PGLs syndrome and mutation in SDHB, SDHC, or SDHD genes [15]. PGLs in the thoracoabdominal region are associated with SDHB mutation whereas the head and neck PGLs are associated with SDHD. The cases reported with SDHB mutation have shown more aggressive behaviour with increased recurrence rates [14].

Among the studies in the literature, a multi-institutional Korean study published by Park et al. has studied 52 cases of PHEO/PGLs retrospectively. They found that the patients with SDHB deficiency are typically young with a mean age of 43 years in comparison with intact SDHB. Regarding tumour size, cases of SDHB deficiency were larger than those with intact SDHB (mean size: 4.5 cm and 1.9 cm respectively). Also, they noticed more instances of lymphovascular invasion with cases of SDHB deficiency. During the follow-up, two cases of SDHB deficiency developed metastasis to lymph nodes and bone [15]. This is in accordance with our findings in the current case. Regarding the malignant behaviour of PHEO/PGLs, metastasis was reported in the bone, lungs, liver, and lymph nodes within three years [1].

## Conclusions

We reported a rare case of a young female with concurrent adrenal PHEO and urinary bladder PGL with positive genetic testing for SDHB mutation. Both tumours were treated surgically; however, the patient ultimately developed recurrence, rapid progression, and metastasis. All secondary modalities were unsuccessful, and the patient was referred for palliative treatment and eventually lost to follow-up. PGL should be considered in the differential diagnosis of bladder tumours. Although PGLs are more commonly associated with sporadic syndromes, gene mutations should be tested as SDHB gene. Therefore, these patients need follow-up and genetic counselling.

## References

[1] Jochmanova I, Wolf KI, King KS (2017). SDHB-related pheochromocytoma and paraganglioma penetrance and genotype-phenotype correlations. J Cancer Res Clin Oncol.

[2] Martucci VL, Pacak K (2014). Pheochromocytoma and paraganglioma: diagnosis, genetics, management, and treatment. Curr Probl Cancer.

[3] Loveys FW, Pushpanathan C, Jackman S (2015). Urinary bladder paraganglioma: AIRP best cases in radiologic-pathologic correlation. Radiographics.

[4] Sugimura R, Kawahara T, Noguchi G (2019). Functional paraganglioma of the bladder: both radiographic-negative and laboratory-negative case. IJU Case Rep.

[5] Giubellino A, Lara K, Martucci V, Huynh T, Agarwal P, Pacak K, Merino MJ (2015). Urinary bladder paragangliomas: how immunohistochemistry can assist to identify patients with SDHB germline and somatic mutations. Am J Surg Pathol.

[6] Martucci VL, Lorenzo ZG, Weintraub M (2015). Association of urinary bladder paragangliomas with germline mutations in the SDHB and VHL genes. Urol Oncol.

[7] Lenders JW, Eisenhofer G, Mannelli M, Pacak K (2005). Phaeochromocytoma. Lancet.

[8] Zimmerman IJ, Biron RE, Macmahon HE (1953). Pheochromocytoma of the urinary bladder. N Engl J Med.

[9] Beilan JA, Lawton A, Hajdenberg J, Rosser CJ (2013). Pheochromocytoma of the urinary bladder: a systematic review of the contemporary literature. BMC Urol.

[10] Williams MD, Tischler AS (2017). Update from the 4th Edition of the World Health Organization Classification of Head and Neck Tumours: Paragangliomas. Head Neck Pathol.

[11] O'Riordain DS, Young WF, Jr. Jr., Grant CS, Carney JA, van Heerden JA (1996). Clinical spectrum and outcome of functional extraadrenal paraganglioma. World J Surg.

[12] Kapetanakis S, Chourmouzi D, Gkasdaris G, Katsaridis V, Eleftheriadis E, Givissis P (2017). Functional extra-adrenal paraganglioma of the retroperitoneum giving thoracolumbar spine metastases after a five-year disease-free follow-up: a rare malignant condition with challenging management. Pan Afr Med J.

[13] Karasek D, Shah U, Frysak Z, Stratakis C, Pacak K (2013). An update on the genetics of pheochromocytoma. J Hum Hypertens.

[14] Amar L, Bertherat J, Baudin E (2005). Genetic testing in pheochromocytoma or functional paraganglioma. J Clin Oncol.

[15] Park S, Kang SY, Kwon GY (2017). Clinicopathologic characteristics and mutational status of succinate dehydrogenase genes in paraganglioma of the urinary bladder: a multi-institutional Korean study. Arch Pathol Lab Med.

